# Supplementary material for: Effect of intraoperative Hartmann’s versus hypotonic solution administration on FLACC pain scale scores in children: A prospective randomized controlled trial
Source: PLoS One. 2020 Mar 19;15(3):e0230556. doi: 10.1371/journal.pone.0230556 (PMC7082008; doi:10.1371/journal.pone.0230556)
Supplement: S1 File — (DOC) [file pone.0230556.s002.doc]

| 임상연구 계획서 | |
| --- | --- |
| (1)임상연구의 명칭 및 단계 | Nuss Bar 제거술을 받는 소아에서 1:2DS 투여가 회복실에서 통증 및 과민성에 미치는 영향 ( Impact of glucose containing hypotonic solution on post operative irritability in pediatric patients with Nuss bar removal. ) |
| (2)임상연구 실시기관명 및 주소 | 가톨릭 대학교 서울성모 병원  서울특별시 서초구 반포동 505 가톨릭대학교 서울성모병원 |
| (3)연구의 책임자 및 담당자/공동연구자 성명, 직위, 소속 | 연구책임자 및 담당자: 김미현/이형묵  소속: 가톨릭 대학교 서울성모 병원 마취통증의학교실  직위: 임상조교수 / 임상강사 |
| (4)의뢰자(기관)명 및 주소 | 해당사항 없음 |
| (5) 임상연구의 목적 | 1. 수술 중 포도당 포함 저장성 수액 (1:2 dextrose solution) 투여로 인한, 저나트륨 혈증 및 고혈당의 발생 유무를 확인하고, 전신마취 회복 후, 과민성 유발 및 통증 반응 강화 여부에 대하여, 회복실에서 FLACC score를 측정하여 확인한다. |
| 임상연구의 배경 | 본원에서는 소아 수술시 1:2 dextrose solution을 기본적인 수액으로 사용하고 있다. 최근 연구에서는, 지속적인 저장성 수액 투여가 저나트륨 혈증 발생을 유발하고, 심한 경우 뇌부종 혹은 뇌탈출을 유발하여, 과민성 유발, 경련 발생 및 사망까지 발생 할 수 있음이 보고 되어 있으며, 이러한 효과는 술 중 및 술 후 항이뇨호르몬의 이상 분비를 통해 강화되는 것으로 알려져 있다. 또한 술 중 고혈당은 허혈성 뇌손상의 발생에 영향을 미치는 것으로 알려져 있다. 하지만, 술 후 지속적인 포도당 포함 저장성 수액 투여의 위험성에 대해서는 많은 연구가 발표되어 있으나, 수술 중 발생하는 저나트륨혈증 및 고혈당과 관련된 전신 마취 직후의 과민성 증가 여부에 대한 정량적인 연구는 시행된바 없다. |
| (6) 임상연구 제품 | 1. 사용 의약품 : 1:2 dextrose solution 500cc, Hartmann’s solution 500cc 2. 약제비 부담 주체 : 수술 중 필수적으로 사용하는 수액으로 환자가 부담함 3. 연구에 사용되는 의약품은 마취과 간호사에 의해 관리되며, 수술실 내 마취과 준비실에 보관되어 있음. |
| (7) 대상질환 | 오목 가슴 치료를 위해 흉부에 삽입된 Nuss bar를 제거하는 수술을 받는 환아. |
| (8) 피험자의 선정기준, 제외기준, 목표한 피험자의 수 및 그 근거 | 1) 선정기준  연구 대상 기관에서 IRB 통과 후 부터, 1년간 오목가슴 교정술시행을 위해 삽입한 Nuss bar 제거 수술 받는 3~10세 환자.  2) 제외기준: 오목가슴 이외에 다른 질환을 가지고 있는 환자는 제외함.  3) 피험자 수: 40명  수술 중 저장성 수액 투여 후 혈중 나트륨 농도 변화를 관찰한  과거 연구에서는 변화량이 2mEq/L +-2.0 로,  power 0.8 수준으로 결과 도출을 위해서는 각 군당 20명 이상이 필요함.  수술 중 수액 투여로 인한 수술 후 통증 및 과민성 변화를  소아에서 정량적으로 측정한 연구는 발견할 수 없었음 |
| (9) 임상연구의 기간 | IRB 승인 후 1년 |
| (10)임상연구의 방법 | 1. 1:2 Dextrose solution 투여 군과 Hartmann’s solution 투여 군을 block randomization method를 이용하여 배정한다. 2. 마취 시작시 금식으로 인한 체내 수분 부족분을 보충한다. 3. 수액 보충 후 혈액 가스 검사를 시행한다. 4. 수술 중 수액 투여량은 정해진 규칙( 유지용량과 금식기간 소실 보충량의 합을 투여하며, 유지용량은 체중 10kg 까지는 4cc/kg/hr, 체중 10~20kg는 2cc/kg/hr, 체중 20kg 이상은 1cc/kg/hr를 투여하고, 보충량은 금식시간 전체 유지용량의 절반을 수술 초기 1시간, 이후에는 금식기간 전체 유지용량의 1/4을 1시간동안 투여한다. )에 의거해 투약한다. 5. 전신마취 종료 시점에서 혈액 가스 검사를 시행한다. 6. 회복실 퇴실 직전, 혹은 진통제 투여가 필요한 경우, 진통제 투여 직전, FLACC score를 측정한다. |
| (11) 관찰항목, 임상검사항목 및 관찰 검사 방법 | 1) 수술 전 검사한 혈중 나트륨, 혈당 농도를 전자의무기록을 통해 확인  2) 전신 마취 시작 및 종료시 혈액 가스 검사를 통한, 혈중 나트륨, 혈당, pH, 락테이트 농도 측정  3) 회복실에서 FLACC score 측정  4) 키, 몸무게, 수술일, 나이, 성별, 수술 전 수액 투여시간, Nuss Bar 개수 ,Nuss Bar 삽입기간을 전자의무기록 및 면담을 통해 확인  5) 회복실에서 오심 및 구토 여부, 진통제 투여 여부 |
| (12) 중지 및 탈락 기준 | 1) 동맥혈 검사에서 혈중 농도 125 mEq/L 이하의 저나트륨 혈증  2) 동맥혈 검사에서 혈중 농도 150 mEq/L 이상의 고나트륨 혈증  3) 동맥혈 검사에서 혈중 농도 80 mg/dL 이하의 저혈당  4) 동맥혈 검사에서 혈중 농도 300mg/dL 이상의 고혈당 |
| (13) 효과 평가기준,평가방법, 해석방법(통계분석방법) | 1) 1:2DS 수액을 투여한 집단과 하트만 수액을 투여한 집단 사이에, 전신마취 유도 후 (T1) 및 전신마취 회복 직전(T2) 의 혈중 나트륨, 혈당, 락테이트 농도의 차이가 발생하는지 T-test를 통해 비교한다. (p<0.05)  2) 회복실에서 FLACC score를 측정하여 Mann-Whitney U test로 두 집단 사이에 차이가 있는지 확인한다.(p<0.05) 회복실에서 의식 회복이 완전하지 않은 경우가 흔하게 있어, 설문을 통해 확인 가능한 VAS, NRS 등을 사용하지 않고, 관찰로 판단 가능한 FLACC score를 사용하여, 통증 정도를 사정한다. |
| (14) 부작용을 포함한 안전성의 평가기준 ,평가방법 및 보고방법 | 수액 투여중 혈액가스 검사를 통해, 수액 투여로 발생할 수 있는 부작용을 확인할 수 있다. 중지 기준에 해당하는 혈액 검사 결과 확인 후, 연구 담당자와 상의하여, 아래와 같이 처치한다.   1. 저혈당 ( < 80mg/dL ) : 5% DW 1~2cc/kg infusion 후 30분 뒤 재 확인하고, 교정되지 않은 경우, 반복 투여 한다. 2. 고혈당 ( > 300mg/dL ) : regular insulin 2u iv 후 30분 뒤 재 확인 하고, 교정되지 않은 경우, 반복 투여 한다. 3. 저나트륨혈증 ( < 125mEq/L ) : 수술 중지하고, normal saline으로 수액 교체. 3% NaCl 투여하면서, 130mEq/L를 목표로, 0.5mEq/L/hr 이하 속도로 교정한다. 4. 고나트륨혈증 ( >150mEq/L ) : 0.45 NaCl로 수액 교체, 145 mEq/L 를 목표로, 0.5mEq/L/hr 이하 속도로 교정한다.   수술 종료시 까지 교정되지 않을 시, 수술실 회복실에서 다시 사정하여, 교정을 지속하고, 해당 임상과에 통보하여, 지속적인 처치가 가능하도록 한다.  이외 중대한 이상반응, 예상하지 못한 문제 발생 시 필수적으로, 책임연구자가 IRB에 보고한다.  본 연구에 사용할 수액의 종류 및 투여량은 일반적으로 본원에서 수술 중 사용되고 있는 수준으로, 상술한 부작용과 본 연구 사이의 인과관계는 매우 낮으며, 피해자 보상이 필요하지 않다. |
| (15) 피험자의 안전보호에 대한 대책  (별표 4) 연구자의 서약서 첨부 | 환자 증례 자료 수집을 위한 병원 등록번호는 증례 수집 후 연구 번호로 변환하여 임상 연구에 사용되며, 병원 등록번호가 기재되어 있는 연구 자료는 암호가 걸려있는 컴퓨터 파일로 책임연구자가 별도 보관한다. (서약서 별첨)  만 6세 이하의 소아는 부모 혹은 법정 대리인에게 동의서를 취득하며, 만 7세 이상의 소아는 소아용 동의서를 이용해 부모와 함께 소아의 동의를 취득한다. |
| (16) 증례기록서 양식(별첨할 것) | 별첨 |
| (17) 기타 임상연구를 안전하고 과학적으로 실시하기 위하여 필요한 사항 | 해당사항 없음 |
| (18) 해당연구의 근거가 되는 임상문헌(참고 문헌) | 별첨 |

- 해당사항이 없는 항목은 ‘해당사항 없음’으로 기재해 주십시오.
- 임상시험/연구와 관련하여 보충 설명/자료가 필요한 경우 별첨하여 주시기 바랍니다.
