# Supplementary material for: Effect of intraoperative Hartmann’s versus hypotonic solution administration on FLACC pain scale scores in children: A prospective randomized controlled trial
Source: PLoS One. 2020 Mar 19;15(3):e0230556. doi: 10.1371/journal.pone.0230556 (PMC7082008; doi:10.1371/journal.pone.0230556)
Supplement: S2 File — (DOC) [file pone.0230556.s003.doc]

| Clinical Trial Plan | |
| --- | --- |
| (1) Clinical trial title and phase | Impact of glucose-containing hypotonic solution on postoperative irritability in pediatric patients with Nuss bar removal |
| (2) Lead investigation site and address | Seoul St. Mary’s Hospital, College of Medicine, The Catholic University of Korea, 222, Banpo-daero, Seocho-gu, Seoul, 06591, Republic of Korea |
| (3) Chief Investigator and principal investigator/collaborator names, positions, and affiliations | Chief and principal investigators: Mihyun Kim/ Hyungmook Lee  Affiliation: Department of Anesthesiology and Pain Medicine, The Catholic University of Korea, Seoul St. Mary’s Hospital  Position: Clinical assistant professor / Clinical lecturer |
| (4) Sponsor (institution) and address | Not applicable |
| (5) Research objective | 1. This study aimed to investigate whether intraoperative administration of a glucose-containing hypotonic solution (1:2 dextrose solution) caused hyponatremia or hyperglycemia, and whether it caused hypersensitivity or increased pain response during postoperative recovery from anesthesia by measuring FLACC scores in the recovery room. |
| Research background | At the investigation site, 1:2 dextrose solution is used as standard for fluid administration in pediatric surgery. In a recent study, continuous hypotonic fluid administration was reported to be capable of causing hyponatremia and, in severe cases, cerebral edema or brain herniation, hypersensitivity, convulsions, and even death. These effects are known to be enhanced by abnormal intraoperative or postoperative secretion of vasopressin. In addition, intraoperative hyperglycemia is known to affect the risk of ischemic brain injury. However, although there have been numerous studies on the risks of continuous intraoperative administration of glucose-containing hypotonic fluids, there have not been any quantitative studies on whether or not hypersensitivity is increased immediately after general anesthesia in association with intraoperative hyponatremia and hyperglycemia. |
| (6) Trial products | 1. Drugs used: 1:2 dextrose solution 500cc, Hartmann’s solution 500cc 2. Responsibility for drug costs: since these solutions are used mandatorily during surgery, the costs were borne by the patients. 3. The drugs used in the trial are managed by nurses in the Department of Anesthesiology, and are stored in the anesthesia preparation room within the operating theater. |
| (7) Targeted condition | Pediatric patients who had undergone surgery for removal of a Nuss bar inserted into the chest to treat pectus excavatum |
| (8) Participant selection and exclusion criteria, targeted number of participants, and related evidence | 1) Inclusion criteria  For 1 year after approval by the IRB at the investigation site, 3–10-year-old patients who underwent surgery for removal of a Nuss bar inserted for correction of pectus excavatum  2) Exclusion criteria: patients with illnesses other than pectus excavatum were excluded.  3) Number of participants: 40 persons  In previous studies observing changes in blood sodium concentration after intraoperative administration of a hypotonic solution, the size of changes was 2 ± 2.0mEq/L. Thus, in order to derive results with a power of 0.8, 20 patients were required per group.  No previous studies were found that had quantitatively measured changes in postoperative pain and hypersensitivity due to intraoperative fluid administration in pediatric patients. |
| (9) Clinical trial duration | 1 year after approval by the IRB |
| (10) Research method | 1. Patients will be allocated to a 1:2 Dextrose solution group or a Hartmann’s solution group using a block randomization method. 2. At the start of anesthesia, fluids will be supplemented for dehydration due to prior fasting. 3. After fluid supplementation, blood gas analysis will be performed. 4. The volume of intraoperative fluids will be determined by predefined rules (the volume administered is equal to the sum of the maintenance dose and the supplementation dose for loss during fasting; the maintenance dose is 4cc/kg/hr up to 10kg body weight, 2cc/kg/hr for body weight between 10–20kg, and 1cc/kg/hr for body weight over 20kg; the supplementation dose is equal to half the maintenance dose for the fasting period during the first hour of surgery, followed by 1/4 of the maintenance dose for the fasting period for 1 hour. 5. Blood gas analysis will be performed upon stopping general anesthesia. 6. FLACC score will be measured immediately before discharge from the recovery room or, in cases requiring analgesic administration, immediately before analgesic administration. |
| (11) Observed variables, clinical test variables, and observational test methods | 1) Preoperative blood sodium and blood glucose levels will be checked via electronic medical records  2) In blood gas analysis at the start and end of general anesthesia, blood sodium, blood glucose, pH, and blood lactate will be measured.  3) FLACC score will be measured in the recovery room.  4) Height, body weight, date of surgery, age, sex, duration of preoperative fluid administration, Nuss bar number, and duration of Nuss bar insertion will be checked via electronic medical records and interview.  5) Nausea and vomiting and the use of analgesics will be recorded in the recovery room. |
| (12) Stoppage/dropout criteria | 1) Hyponatremia (blood sodium <125 mEq/L) in arterial blood tests  2) Hypernatremia (blood sodium >150 mEq/L) in arterial blood tests  3) Hypoglycemia (blood glucose <80 mg/dL) in arterial blood tests  4) Hyperglycemia (blood glucose >300 mg/dL) in arterial blood tests |
| (13) Efficacy assessment criteria, assessment methods, and interpretation (statistical analysis) | 1) T-tests will be performed to examine whether there are any differences between the 1:2DS group and the Hartmann’s solution group in blood sodium, blood glucose, and blood lactate after anesthesia induction (T1) or immediately before anesthesia recovery (T2) (p<0.05).  2) FLACC score will be measured in the recovery room, and a Mann-Whitney U test will be performed to examine whether there is a difference between the two groups (p<0.05). Since recovery of consciousness is often not complete in the recovery room, FLACC score, which is judged by observation, was used to evaluate pain instead of questionnaire-based methods, such as VAS and NRS. |
| (14) Safety assessment criteria, including adverse effects, assessment methods, and reporting methods | Potential adverse effects of fluid administration can be identified via blood gas analysis during fluid administration. In the event of blood test results pertaining to the stoppage criteria, the principal investigator will be consulted and the following measures will be taken:   1. Hypoglycemia ( < 80mg/dL ) : After infusion of 1–2cc/kg of 5% DW, blood glucose is checked again 30 minutes later, and if hypoglycemia remains uncorrected, the dose is repeated. 2. Hyperglycemia ( > 300mg/dL ) : After IV infusion of 2u of regular insulin, glucose is checked again 30 minutes later, and if hyperglycemia remains uncorrected, the dose is repeated. 3. Hyponatremia ( < 125mEq/L ) : Surgery is stopped, and fluids are replaced with normal saline. 3% NaCl is administered to correct blood sodium levels to 130mEq/L, at a rate of <0.5mEq/L/hr. 4. Hypernatremia ( >150mEq/L ) : Fluids are replaced with 0.45 NaCl solution to correct blood sodium levels to 145mEq/L, at a rate of <0.5mEq/L/hr.   If adverse effects cannot be corrected before completing surgery, the patient’s state will be re-assessed in the recovery room, correction will be continued, and the relevant clinical department will be informed to enable continual management.  In the event of any other serious adverse effects or unexpected problems, these will be reported to the IRB by the chief investigator without fail.  The types and doses of fluids to be used in this trial are already used commonly at the investigation site. Thus, there is very little causal association between this trial and the adverse effects described above, and there is no need for participant compensation. |
| (15) Measures to ensure participant safety  (Appendix 4) Participant agreement form | The hospital registration number for collecting patient case data will be converted to a trial reference number after case selection for use in the clinical trial, and any research data containing the hospital registration number will be stored separately by the chief investigator in an encrypted computer file (the agreement form has been attached separately).  For children aged 6 years or younger, written consent was obtained from the child’s parents or a legal representative. For children aged 7 years or older, a pediatric consent form was used to obtain consent from the child together with their parents. |
| (16) Case record form (to be attached separately) | Attached separately |
| (17) Other requirements for safe and scientific completion of the clinical trial | Not applicable |
| (18) Clinical literature providing evidence for the clinical trial (references) | Attached separately |
