# Supplementary material for: Effect of intraoperative Hartmann’s versus hypotonic solution administration on FLACC pain scale scores in children: A prospective randomized controlled trial
Source: PLoS One. 2020 Mar 19;15(3):e0230556. doi: 10.1371/journal.pone.0230556 (PMC7082008; doi:10.1371/journal.pone.0230556)
Supplement: Attachment — Submitted filename: Response to Reviewers.docx [file pone.0230556.s004.docx]

Response to Reviewers

To Editor :

As per the journal’s editorial policy, please include in the Methods section of your paper:
a) your reasons for your delay in registering this study (after enrolment of participants started);

- It was because of miscommunication between authors. We thought the trial was registered but it was not. After we found it, we registered the study at once. ( line 65-66 )

b) confirmation that all related trials are registered by stating: “The authors confirm that all ongoing and related trials for this drug/intervention are registered”.

- We added the suggested sentence. ( line 64-65 )

Please also ensure you report the date at which the ethics committee approved the study as well as the complete date range for patient recruitment and follow-up in the Methods section of your manuscript.

- We added an arrival date by the ethics committee. ( line 62 )
- We added the complete date range for patient recruitment. Follow-up of the patient was not necessary because the study was done only in the operation room and the post anesthesia care unit. We also mentioned it. ( line 69-70)

The [PLOS Data policy](http://www.plosone.org/static/policies.action#sharing) requires authors to make all data underlying the findings described in their manuscript fully available without restriction, with rare exception (please refer to the Data Availability Statement in the manuscript PDF file). The data should be provided as part of the manuscript or its supporting information, or deposited to a public repository. For example, in addition to summary statistics, the data points behind means, medians and variance measures should be available. If there are restrictions on publicly sharing data—e.g. participant privacy or use of data from a third party—those must be specified.

- We deposited the data to public repository.

(DOI : http://dx.doi.org/10.17632/63mbdj7dyd.2 )

To mask the patients’ information, We omitted the operation date and rearranged the order of data.

To : Reviewer #1

General It would simplify the presentation if ‘Dextrose solution group’ and ‘Hartmann’s solution’ could be given acronyms, say DEXT and HART, which would then simplify the subsequent text.

- We assign “HYPO” to 1:2 Dextrose solution group and “ISO” to Hartmann’s solution group. We think it is more appropriate to use “HYPO” instead of “DEXT” because the primary interest of the current study is sodium.

32 Move both (n = 20) to Results section of the Abstract

- We modified the manuscript as suggested by Reviewer #1 (line 34)

34 Very important that the difference in FLACC mean scores between the groups is quoted together with the 95% CI

- We add the difference in FLACC mean scores between the groups together with the 95% CI both in abstract and result section as suggested by Reviewer #1 ( line 35, line 146-147 )

34 Usual to quote p-values to two significant figures only – here 0.044

- We modified the manuscript as suggested by Reviewer #1 ( line 35 )

36 Very important that the difference in fentanyl dose mean scores between the groups is quoted together with the 95% CI

- We add the difference in fentanyl dose mean scores between the groups together with the 95% CI as suggested by Reviewer #1 ( line 38, 154 )

36 Usual to quote p-values to two significant figures only – here 0.042

- We modified the manuscript as suggested by Reviewer #1 ( line 37,154 )

Table 1 Although it is not wrong here to quote mean and standard deviation, here it is usually more informative for the reader to quote mean and range here.

- We substitute range for standard deviation as suggested by Reviewer #1. ( line 99 )

We remove preoperative plasma sodium & glucose value. We moved them into table 3. We add Total anesthesia time and infused fluid per bodyweight into the table.

We made a correction for note of the table. The values in the table are median, not mean.

134 -160 Much of this would be better presented in tabular form. As it is, it makes very confusing reading.
General I have counted 18 statistical tests reported in this paper. That is one for every two patients recruited. This is far too many, testing should be confined to the main outcome measures FLACC and Fentanyl and possibly one or two more. The rest of the comparisons should be only made descriptively.

- We rewrite most of the results section to follow Reviewer #1’s recommendation. We also made a table 3 for plasma sodium and glucose level. ( line 162-175 )

Figs 4 & 5 What is more important than the above comment is that these Figures and their associated text in the Results section should be given more prominence and moved to near the beginning of the results section. To me this is the major flaw in this otherwise good paper.

- We re-arrange the mentioned paragraph into the beginning of the results section as suggested by Reviewer #1. Fig 4 & 5 are now Fig 2 & 3. ( line 144-160 )

To : Reviewer #2

The authors appear to stress that the main difference between the solutions is the dextrose ratios. But are there other constituents of the solutions (NaCl, calcium, etc) which might account for the observed difference. This may need to be included in the discussion.

- The constitution of the two infusions is explained in the new table, Table 1.

The main difference between the solutions is the concentration of sodium and we discuss the risk of the low sodium concentration solution in Discussion section.

The authors should state and describe more clearly which solutions are isotonic and which are hypotonic, and label them as such in tables and graphs.

- We assign “HYPO” to 1:2 Dextrose solution group and “ISO” to Hartmann’s solution group.

Also we assign “hypotonic dexterous solution” to 1:2 Dextrous solution and “isotonic solution” to Hartmann’s solution.

We change or add labels in tables and graphs as recommended by reviewer #2

There are multiple other outcomes of interest. However there is no indication in the stat methods section that there was (or wasn't) an adjustment for multiple comparisons.
Whichever it was should be stated clearly.

- We used repeated measure 2-way ANOVA for multiple comparisons. We re-write the paragraph for statistics and include a sentence as follow :

“For plasma sodium and glucose level at T0, T1, and T2, we performed a repeated measures 2-way ANOVA to determine differences between the groups and over time.” ( line 121-123 )

To :Reviewer #3

Please list the constitution (sodium, potassium, glucose concentration, and etc) of the two infusions in new table.

- The constitution of the two infusions is explained in the new table, Table 1.

( line 99 )

To: Reviewer #4

In both the abstract and the manuscript, the rationale for why 1 – 2% dextrose solution was being used is poorly explained. There really is no rational for it. It is sufficient to say that it has been common practice to administer hypotonic fluids to children intraoperatively based on previous recommendation by Holiday-Segar, or something like that. It is really based on tradition and no sound physiology.

- To follow the recommendation from reviewer #4, we add sentences and references as below.

“It is based on a traditional recommendation by Holiday-Segar[6]. However, hypotonic solution could decrease plasma sodium level during induction of general anesthesia[7].” ( line 50-52 )

Reference 5 is not listed and I doubt it exists.

- We fix the citation error. ( line 50 )

Reference 6 is an outlier and is not reflective of the majority of studies.

- We remove the reference 6 and related sentences as recommended by reviewer #4

In both the abstract and the methods, the design of the study is not explicitly stated. It should explicitly state to compare an intraoperative hypotonic solution with dextrose to Hartmann’s solution to evaluate the FLACC scale score and the change in serum sodium and glucose immediately prior to and following surgery. Or something like that.

- We add a sentence as below to follow reviewer #4’s suggestion.

“We compared the effects of an intraoperative 1:2 dextrose solution (hypotonic dextrous solution) with Hartmann’s solution(isotonic solution) on the postoperative FLACC scale scores and the change in plasma sodium and glucose immediately prior to and following surgery.” ( line 77-79 )

Please explain clearly what 1:2 dextrose solution is. What is the dextrose concentration of this fluid and what is the sodium and potassium concentration of this fluid.

- We made a table for the composition of study fluids at Table 1 in Patients and Methods. ( line 99 )

The TO, T1 an T2 time points along with what is being measured needs to occur earlier in the methods and be more explicit.

- We re-write a related sentence and move it more front : ( line 80-82 )

“Plasma sodium and glucose levels were assessed the day before surgery (T0; preoperative baseline values), immediately prior to the surgery (T1), and at the end of surgery before awakening the patient (T2).”

It is not clear what the term waiting room mean. Better to say the preoperative waiting room and recovery room.

- We change them “wating room” to “preoperative waiting room” as suggested by Reviewer #4. ( line 94,95, 106, 226 )

The limits of 125 – 150 are too broad. There were probably no patient with a serum sodium of between 125 – 130. The were no patients with a serum sodium < 130 with the lowest sodium in the group 132. Probably better to change this to 130 – 150. Everyone may have had a normal serum sodium at T0, so you could also say 135 -145 if it was true.

- When we conduct another clinical trial, we will narrow the range following Reviewer #4's suggestion.

The statement about desalinization does not make any sense and should be removed. Saline is no more likely to call desalinization then a more hypotonic fluids and is not a reason not to use it.

- We removed the entire sentence about salinization and the associated reference as suggested by reviewer #4.
