# Supplementary material for: Effect of intraoperative Hartmann’s versus hypotonic solution administration on FLACC pain scale scores in children: A prospective randomized controlled trial
Source: PLoS One. 2020 Mar 19;15(3):e0230556. doi: 10.1371/journal.pone.0230556 (PMC7082008; doi:10.1371/journal.pone.0230556)
Supplement: Attachment — Submitted filename: Response to Reviewers.docx [file pone.0230556.s005.docx]

Response to Reviewers

To editor:

To enhance the reproducibility of your results, we recommend that if applicable you deposit your laboratory protocols in protocols.io, where a protocol can be assigned its own identifier (DOI) such that it can be cited independently in the future. For instructions see: <http://journals.plos.org/plosone/s/submission-guidelines#loc-laboratory-protocols>

- Since the protocol of this study is relatively simple and easy to follow, we decided not to deposit the protocols in protols.io.

To reviewer #1:

Table 3 Title should be amended by adding: ‘Mean and SD of plasma …’

Table 3 CI for plasma quote to 2 decimal places only

Table 3 P-values to 2 decimal places: Amend to 0.99 (although strictly 1) and 0.73

- We mended table 3 as recommended by reviewer #1. ( line 136 in Manuscript )
